# The Structure of a Conserved Telomeric Region Associated with Variant Antigen Loci in the Blood Parasite *Trypanosoma congolense*

**DOI:** 10.1093/gbe/evy186

**Published:** 2018-08-25

**Authors:** Ali Hadi Abbas, Sara Silva Pereira, Simon D'Archivio, Bill Wickstead, Liam J Morrison, Neil Hall, Christiane Hertz-Fowler, Alistair C Darby, Andrew P Jackson

**Affiliations:** 1 Centre for Genomic Research, Biosciences Building, Liverpool, United Kingdom; 2 Department of Pathology, Faculty of Veterinary Medicine, University of Kufa, Najaf, Iraq; 3 Department of Infection Biology, Institute of Infection and Global Health, University of Liverpool, United Kingdom; 4 School of Life Sciences, University of Nottingham, United Kingdom; 5 Department of Infection and Immunity, The Roslin Institute, Easter Bush, Edinburgh, United Kingdom; 6 Earlham Institute, Norwich Research Park, Norwich, United Kingdom

**Keywords:** *Trypanosoma congolense*, variant surface glycoprotein, expression site, telomere, antigenic variation, ESAG

## Abstract

African trypanosomiasis is a vector-borne disease of humans and livestock caused by African trypanosomes (*Trypanosoma* spp.). Survival in the vertebrate bloodstream depends on antigenic variation of Variant Surface Glycoproteins (VSGs) coating the parasite surface. In *T. brucei*, a model for antigenic variation, monoallelic *VSG* expression originates from dedicated *VSG* expression sites (VES). *Trypanosoma brucei* VES have a conserved structure consisting of a telomeric *VSG* locus downstream of unique, repeat sequences, and an independent promoter. Additional protein-coding sequences, known as “Expression Site Associated Genes (ESAGs)”, are also often present and are implicated in diverse, bloodstream-stage functions. *Trypanosoma congolense* is a related veterinary pathogen, also displaying *VSG*-mediated antigenic variation. A *T. congolense* VES has not been described, making it unclear if regulation of *VSG* expression is conserved between species. Here, we describe a conserved telomeric region associated with *VSG* loci from long-read DNA sequencing of two *T. congolense* strains, which consists of a distal repeat, conserved noncoding elements and other genes besides the *VSG*; although these are not orthologous to *T. brucei* ESAGs. Most conserved telomeric regions are associated with accessory minichromosomes, but the same structure may also be associated with megabase chromosomes. We propose that this region represents the *T. congolense* VES, and through comparison with *T. brucei*, we discuss the parallel evolution of antigenic switching mechanisms, and unique adaptation of the *T. brucei* VES for developmental regulation of bloodstream-stage genes. Hence, we provide a basis for understanding antigenic switching in *T. congolense* and the origins of the African trypanosome VES.

## Background

Many eukaryotic genomes display a distinct elaboration of the subtelomere, the region between chromosomal cores and the telomeres, through the accumulation of species-specific, contingency gene families (Das et al. 2008; Brown et al. 2010). This is especially true for parasites, in which multicopy gene families are often implicitly associated with virulence and mechanisms for pathogenesis and immuno-modulation (Rehmeyer et al. 2006; Sargeant et al. 2006; Hayashida et al. 2013; Reid 2015). As such, parasite subtelomeric regions assume special significance as “contingency regions”, in which genes may duplicate and diversify through recombination, and where the gene expression may be precisely regulated through positional silencing and epigenetic modification (Barry et al. 2003; Kissinger and DeBarry 2011). 

African trypanosomes (*Trypanosoma* spp.) are unicellular hemoparasites that cause African trypanosomiasis, a disease of humans and animals transmitted by biting tsetse flies. They provide an excellent example of how the subtelomere is adapted for the expression of contingency genes (Jackson 2016). In their vertebrate host, trypanosomes multiply extracellularly in the bloodstream, passing readily to peripheral tissues such as skin and adipose tissue, and ultimately to the central nervous system. The parasites are able to maintain a chronic infection resulting in recurrent fever, anemia, neurological dysfunction, and then death if untreated. African trypanosomes evade host antibodies through antigenic variation, a strategy of modulating their immunogenic cell surface antigens that many pathogens have converged upon (Deitsch et al. 2009; Vink et al. 2012). 

Antigenic variation occurs in African trypanosomes through sequential replacement of the variant surface glycoprotein (VSG), which coat the parasite surface during its infective, metacyclic and bloodstream life stages. VSG are encoded by a contingency gene family comprising several thousand genes that are subject to frequent duplication and transposition (Berriman 2005; Jackson et al. 2010, 2012). Most *VSG* loci are found on extended subtelomeric regions of megabase chromosomes, which are repetitive and typically lack housekeeping genes (Callejas et al. 2006). The *T. brucei* genome also includes ∼100 “minichromosomes” that typically include one or 2 telomeric *VSG* genes each (Williams et al. 1982; Zomerdijk et al. 1992; Robinson et al. 1999; Cross et al. 2014), preceded by 70 bp repeat regions (Liu et al. 1983; Sloof, Bos, et al. 1983; Cross et al. 2014), and either side of a central core of 177 bp repeats (Wickstead et al. 2004). The parasite infrapopulation consists of multiple clones, each expressing a distinct *VSG* gene. Through periodic *VSG* switching and frequency-dependent expansion of parasite clones expressing new variants, the parasite population can evade the humoral immune response indefinitely. 

Our understanding of how *VSG* expression is regulated comes almost exclusively from the model species, *Trypanosoma brucei* (Horn 2014). Monoallelic expression is achieved by location of the active *VSG* gene within one of several telomeric *VSG* expression sites (VES), to which *VSG* transcription is strictly limited (Glover et al. 2013), coupled with genome-wide transcriptional repression of all other VES. Antigenic switching involves either the replacement of the active *VSG* by an alternative through ectopic gene conversion, or the activation of an alternative VES through epigenetic mechanisms (Rudenko 2011; Duraisingh and Horn 2016). 

In *T. brucei*, there are metacyclic and bloodstream VES. The metacyclic VES, which is active in mammalian-infective, metacyclic-stage parasites inhabiting the tsetse fly mouthparts, has been shown to comprise, moving from 5′ to 3′ end, a 50 bp repeat region that effectively marks the boundary between the expression site and the distal subtelomeric region, a promoter, a region of 70 bp repeat, the *VSG* and a telomeric repeat (Campbell et al. 1984; Lenardo et al. 1984; Cornelissen et al. 1985; Lenardo et al. 1986). The structure of the *T. brucei* bloodstream-VES, which is active while the parasite replicates in the mammalian bloodstream, was fully described through Sanger sequencing of Bacterial Artificial Chromosomes comprising *T. brucei* Lister 427 genomic fragments containing active *VSG* genes (Berriman et al. 2002), and subsequently isolated and sequenced using transformation-associated recombination (TAR) cloning in yeast, using known VES motifs as baits (Becker 2004; Hertz-Fowler et al. 2008). It contains a collection of non-*VSG* genes in addition to the monocistronic structure of the metacyclic-VES. These additional coding genes are known as Expression Site Associated Genes (ESAG1–13); and they are typically derivations of conserved cell surface-located gene families, uniquely associated with *T. brucei* expression sites (Jackson et al. 2013)—a prime example being of SRA, which confers a human infectivity phenotype when present in the bloodstream VES (Van Xong et al. 1998). Comparison of expression sites shows that the order and spatial relationships of coding and noncoding elements is highly conserved across chromosomes and between strains, and also, that expression site sequences frequently recombine, probably through telomeric exchange or smaller gene conversion events (Hertz-Fowler et al. 2008).

Although the *T. brucei* VES is well understood, it is unclear if the same structures and mechanisms exist in other trypanosomes. This matters because AAT is caused by several trypanosome species; *T. brucei* often causes only a small proportion of veterinary disease, whereas *T. congolense* can be the cause of up to 50% of animal trypanosome infections in some locations (Takeet et al. 2013; Mamoudou et al. 2016). Similarly to *T. brucei*, *T. congolense* displays antigenic variation of homologous *VSG* (Majiwa et al. 1985; Strickler et al. 1987), switching between variant antigen types to evade the immune response (Nantulya et al. 1980), with each *T. congolense* infrapopulation predominantly expressing a single active *VSG* (Helm et al. 2009). Majiwa et al. (1985) described antigenic switching in *T. congolense* and showed that *VSG* were expressed from telomeric loci, as in *T. brucei*. Although a VES is assumed to exist in *T. congolense*, the context for *VSG* expression has not been described. In this study, we examined *T. congolense* telomeres for possible expression sites.

Using long-read DNA sequencing on the PacBio platform, it is now possible to capture a large proportion of telomeric ends with appreciable genomic context. Our aim was to survey multiple *T. congolense* telomeric ends from two distinct strains (Tc1/148 and IL3000) to examine if a conserved telomeric region exists in *T. congolense*, homologous to the *T. brucei* VES. We show that telomere-proximal regions in *T. congolense* do display a common organization, which we propose is representative of the VES. As this is topologically similar, but not strictly homologous, to the *T. brucei* VES, it indicates common ancestry and perhaps functional conservation, but also mechanistic divergence.

## Materials and Methods

### Parasite Stocks and Culture

*Trypanosoma congolense* savannah 1/148 (MBOI/NG/60/1-148) procyclic forms, firstly isolated in Nigeria in 1960 (Young and Godfrey 1983), were cultured in modified Eagle’s medium-based modified differentiating trypanosome medium (10% fetal bovine serum, 2 mM glutamine, 10 mM proline) in 25 cm^2^ flasks and incubated at 27 °C, 5% CO_2_. *Trypanosoma congolense* savannah IL3000 blood stage forms, firstly isolated in Kenya in 1966 (Gibson 2012), were cultured in TcBSF-3 media in 24-well plates and incubated at 34 °C in humid incubator of 5% CO_2_ atmosphere to the mid log phase, as described by Coustou et al. (2010). Cultures were harvested when cell number reached approximately 1.2× 10^9^ cells by centrifugation at 1,000 ×g and DNA was extracted from the cell pellet using a Qiagen DNeasy Blood and Tissue Kit, following the manufacturer’s protocol.

### Southern Blot

Whole-chromosome-sized DNAs were prepared in agarose plugs as described in Melville et al. (1998). Cells were harvested from culture medium by centrifugation for 10 min at 1,200×g, washed once in TDB (120 mM NaCl, 5 mM KCl, 15 mM sodium phosphate, 30 mM Tris–HCl, pH 8), reharvested and set into 0.8% low-melting temperature agarose (SeaPlaque GTG, Lonza) at a final concentration of 4× 10^8^ cells/ml. Agarose plugs containing trypanosomes were extracted against 1% *N*-lauroyl sarcosine, 10 mM Tris base, 0.5 M EDTA containing 1 mg/ml proteinase K (Roche) at 50 °C, first at pH 9 then pH 8 for 24 h each. Plugs were dialysed extensively against 10 mM Tris–HCl, 1 mM EDTA before electrophoresis. Pulsed-field gel electrophoresis was carried out in a contour-clamped homogeneous electric field electrophoresis apparatus (CHEF DRIII; Biorad). DNA separation was performed in 1% agarose (SeaKem Gold, Lonza) in 90 mM Tris–borate, 0.2 mM EDTA, pH 8.2, at 12 °C for 42 h with a voltage gradient of 4.6 V cm^−1^ and switching time linearly ramped 10–25 s; 2 × 10^7^ cell equivalents were loaded per lane. Pulsed-field gels were stained for 20 min in 1 μg/ml ethidium bromide before visualization. DNA was then nicked with UV (80 mJ; ∼250 nm wavelength) and transferred to a positively charged nylon membrane (Roche) by capillary transfer in 0.4 M NaOH, 1.5 M NaCl.

Labeled probes for *T. brucei* and *T. congolense* 177 and 369 bp repeats (Sloof, Bos, et al. 1983; Sloof, Menke, et al. 1983; Moser et al. 1989) were amplified from genomic DNA by PCR with the inclusion of Fluorescein-12-dUTP (primers: TAAATGGTTCTTATACGAATG and AACACTAAAGAACAGCGTTG, and CAAAATGGCCAAAAACCGG and CATTTTGGCCCAAAAAGGTG, respectively). The probe for detection of telomeric repeat was synthetic (TTAGGG)_5_ conjugated to fluorescein at the 5′ end. Hybridization was performed overnight in 1% (w/v) sodium dodecyl sulfate, 5% (w/v) dextran sulfate, 10% (w/v) blocking solution (Roche), 750 mM NaCl, 75 mM sodium citrate (pH 7) at 60 °C and washes were performed at 62 °C with a stringency 0.1% (w/v) sodium dodecyl sulfate, 30 mM NaCl, 3 mM sodium citrate (pH 7). Probes were detected with antifluorescein Fab fragments conjugated to alkaline phosphatase (Roche) followed by CPD-star (Sigma-Aldrich). For reprobing, membranes were stripped by washing twice for 10 min with just-boiled 0.3% (w/v) sodium dodecyl sulfate and 0.3 M NaOH, followed by neutralization with 0.5 M Tris–HCl (pH 7).

### DNA Extraction and Sequencing

High Molecular Weight DNA was extracted from 1.2 x 10^9^ cells using a double phenol: chloroform protocol. Cells were centrifuged at 1,500 × g for 10 min and washed in 10 ml cold PBS. Cells were centrifuged at 1,500 × g for 10 min and the supernatant was discarded. The pellet was resuspended in 500 μl PBS and incubated with 6 ml TELT buffer (1.5 M LiCl anhyd, 50 mM Tris–HCl pH 8.0, 62.5 mM EDTA pH 8.0, 4% Triton-X) at room temperature for 5 min. 7 ml of 1:1 phenol:chloroform was added and mixed by inversion for 5 min or until emulsion was formed. The emulsion was centrifuged at 3,000 × g for 5 min and the aqueous phase retained. Two volumes of ethanol were added to the aqueous solution, mixed by inversion, incubated on ice for 10 min and centrifuged at 4,000 × g for 20 min. The pellet was washed in 2 volumes of freeze-cold 70% ethanol, left to air dry at 70 °C for 5 min and then redissolved in 600 μl TE50 (10 mM Tris–HCl pH 8.0, 50 mM EDTA pH 8.0). To remove RNA and or protein contaminants, 150 μg/ml of RNase A was added to the resuspended pellet and incubated for 1 h at 37 °C, and then 300 μg/μl of Proteinase K was added to the solution and incubated for 2 h at 50 °C. After the incubation period, 600 μl 1:1 phenol:chloroform was added and mixed by inversion for 5 min. The solution was centrifuged at 3,000 × g for 5 min and aqueous fraction collected in a 1.5 ml tube. To the aqueous solution, 1 volume of isopropanol and 0.1 volumes of 3 M sodium acetate (NaOAC) were added. The solution was centrifuged at 1,500 × g for 15 min at 4 °C, the pellet washed in 1 ml freeze-cold 70% ethanol, and then redissolved in TE50 (2 μl/10^7^ cells) at 4 °C overnight, without pipetting or mechanically disturbance. A total of 20 kb genomic libraries were prepared from the DNA at the Centre for Genome Research (University of Liverpool) and sequenced on the PacBio SMRT sequencer RSII (Pacific Biosciences, USA).

### Assembly and Annotation

Raw sequence reads were filtered for quality and assembled using the standard PacBio proprietary software Hierarchical Genome Assembly Process 3 (HGAP3) (Chin et al. 2013). HGAP3 was run under default conditions, that is, automatic calculation of minimum seed read length to produce a minimum of 30 ×  genome coverage, and a predicted genome size of 40 Mb (Jackson et al. 2012). The Tc1/148 assembly contained 536 contigs (*n*50 = 421,740 bp) and genome coverage of 70X, whereas the IL3000 assembly produced 1,541 contigs (*n*50 = 156,211 bp) and a genome coverage of 47X. Assembled contigs were annotated using the web server Companion (Steinbiss et al. 2016), employing RATT (Otto et al. 2011) on species mode to transfer corresponding annotation from *T. brucei* 927. Gene finding was also carried out ab initio using AUGUSTUS (Stanke et al. 2004) with a score threshold of 0.7 to make gene prediction more sensitive, and to identify open reading frames not present in the *T. brucei* genome. Finally, all annotated telomeric features were manually curated based on BLASTx protein evidence. The Tc1/148 genome has been deposited at DDBJ/ENA/GenBank under the accession NHO00000000. The *T. congolense* IL3000 genome has been deposited at DDBJ/ENA/GenBank under the accession PQVL00000000.

### Telomere Annotation

Assemblies were screened for the telomeric repeat using Repeat Masker (http://repeatmasker.org). Contigs containing telomeric repeats in the expected, terminal position were then manually inspected for sequence features using Artemis (Rutherford et al. 2000). Open reading frames were initially identified using Artemis (Rutherford et al. 2000) and manually curated. We were interested in identifying sequences that positively associated with the telomere, which might possibly be *T. congolense* specific and repetitive in nature. Because such novel features would not necessarily be detected by either Companion or AUGUSTUS, telomere annotation was performed manually. We examined all open reading frames within telomere-containing contigs >100 bp in length using BLASTn and tBLASTx (Altschul et al. 1990), searching against a database of all *T. congolense* IL3000 and *T. brucei* 927 genes with a significance threshold (E-value) of 10^−4^. The Artemis Comparison Tool (ACT) (Carver et al. 2005), which also applies BLASTn and tBLASTx, was used to compare pairs of telomere-containing contigs and thereby identify homologous, noncoding elements that were present in multiple instances. 

### Phylogenetic Analysis

Contig comparison identified both coding and noncoding sequences conserved across multiple telomere-containing contigs. ClustalW (Larkin et al. 2007) was used to align both nucleotide sequences of four conserved noncoding elements (CNE) of Tc1/148, as well as amino acid sequences of conserved coding regions found in telomere-containing contigs (Fam15, Fam53, DEAH-box RNA helicase, cathepsin B), followed by manual correction where necessary. Maximum likelihood (ML) phylogenies were estimated from amino acid sequence alignments following automatic model selection (Lefort et al. 2017) using PHYML v3.0 (Guindon and Gascuel 2003). IQtree (Nguyen et al. 2015) was used to estimate alternative ML and Bayesian phylogenies from the same amino acid alignments following automatic model selection to corroborate topologies. Robustness was assessed with 100 bootstrap replicates.

Differences in phylogenetic signal between the four CNEs identified across multiple telomere-containing contigs were evaluated by topological comparison. CNEs taken from the same set of contigs should display the same phylogenetic relationships in the absence of any structural rearrangement. We tested this null hypothesis using a Shimodaira–Hasegawa test (Shimodaira and Hasegawa 1999) applied to four cases (CNE1 vs. CNE2, 3 and 4, respectively, and CNE 3 vs. 4). Further comparison of CNE2 and 3 phylogenies was not possible because there were insufficient contigs containing both features. In each case, the log-likelihood value of the optimal ML tree for one CNE (shown on the right in each tanglegram) was compared with the log-likelihood value of a tree constrained to the CNE1 topology (or the CNE3 topology in the last instance) using RaxML (Stamatakis 2014). This produces a *P*-value; significant values (*P*-value <0.05) indicate that optimal and constrained topologies differ to an extent greater than sampling error, which allows the null hypothesis to be rejected and confirms that two trees are significantly different.

### Recombination Tests

To test for evidence of recombination, Phi (Bruen et al. 2006) and Topali (Milne et al. 2009) were applied to quartets (i.e., subsets of four telomere-containing contig sequences) of all contigs containing at least two conserved, noncoding elements (CNE1–4). Quartet alignments were produced with MUSCLE (Edgar 2004) and subsequently manually curated to include only sites common to the first sequence, avoiding excessive gaps. Phi and Topali each execute a sliding window analysis of phylogenetic signal along the alignment to detect changes in optimal tree topology, which are interpreted as evidence for recombination breakpoints. Phi calculates the probability of any recombination breakpoint within the sequence (Bruen et al. 2006). Topali calculates the probability of the three possible phylogenetic topologies along a quartet alignment, and infers the recombination breakpoints as the points where the most probable topology changes (Milne et al. 2009).

## Results

**Fig. 1.**
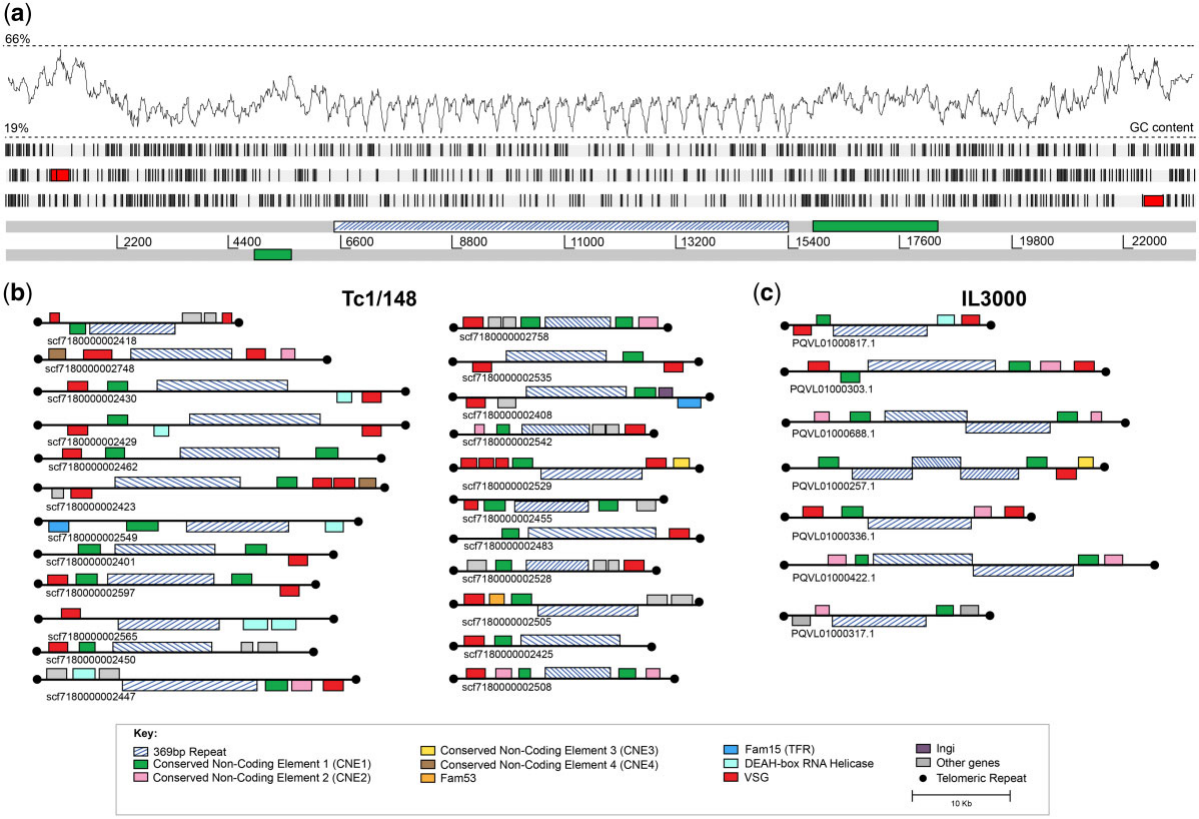
—Minichromosome structure in *T. congolense* 1/148 and IL3000. (*a*) An example Artemis plot of a complete minichromosome from the Tc1/148 genome sequence captured in a single PacBio DNA sequence read. The gray bar represents the DNA sequence, with a scale in base pairs shown below. The three forward codon reading frames are shown above; black vertical lines within the codon reading frames represent stop codons. The positions of conserved coding and noncoding features are shown by the colored boxes on the relevant codon reading frame or DNA, respectively, and according to the key. In this example, the 369 bp repeat is orientated in one direction. Percentage GC, calculated on a sliding window, is plotted at the top. (*b*) Cartoons of all fully sequenced, unique minichromosomes from Tc1/148. Conserved features are drawn on the relevant strand of the DNA sequence (black line). Cartoons are drawn to scale, and aligned at the 5′ end. The direction of shading of the 369 bp repeat region (dark blue) indicates the direction of the repeat sequence, showing that in IL3000 this can occur as a palindrome. Accession codes for the corresponding data contig are given below each case. (*c*) Cartoons of all fully sequenced, unique minichromosomes from IL3000.

The Tc1/148 genome assembly includes 536 contigs, of which 153 contained telomeric repeats at their ends. Of these telomeric contigs, 25 appear to represent complete minichromosome sequences. Figure 1*a* shows an example of the sequence of a complete minichromosome. They possess telomeric repeats at both ends and are between 20,914 and 37,974 bp in length (fig. 1*b*). A further three telomeric sequences were joined in the HGAP3 assembly with TcIL3000 megabase chromosomes 6, 10, and 11 (1.16 Mb, 1.78 Mb, and 1.57 Mb, respectively) (see below for further details). The remaining 125 contigs could not be unequivocally allocated to either mini- or megabase chromosomes because of their size. Assuming that *T. congolense* has a similar number of megabase chromosomes (11) to its sister species *T. brucei* (Melville et al. 1998), we believe that the majority represent partial minichromosomes. The IL3000 genome assembly produced 1541 contigs, of which 128 contained telomeres. Of these, 7 are complete minichromosomes of 21,075–37,994 bp in length (fig. 1*c*). The minichromosome contigs consist of a core 369 bp repeat, flanked by various coding and noncoding elements that are internal to the telomeric repeats (fig. 1*b* and *c*). In the following sections, we define this complex of coding and noncoding sequences as a “conserved telomeric region” associated with *T. congolense* minichromosomes, but perhaps also megabase chromosomes. In at least 15 cases, this conserved region is contained within a single sequencing read, meaning that the overall minichromosome architecture is supported independently of sequence assembly.

### The Conserved Structure of *T. congolense* Telomeric Regions

**Fig. 2.**
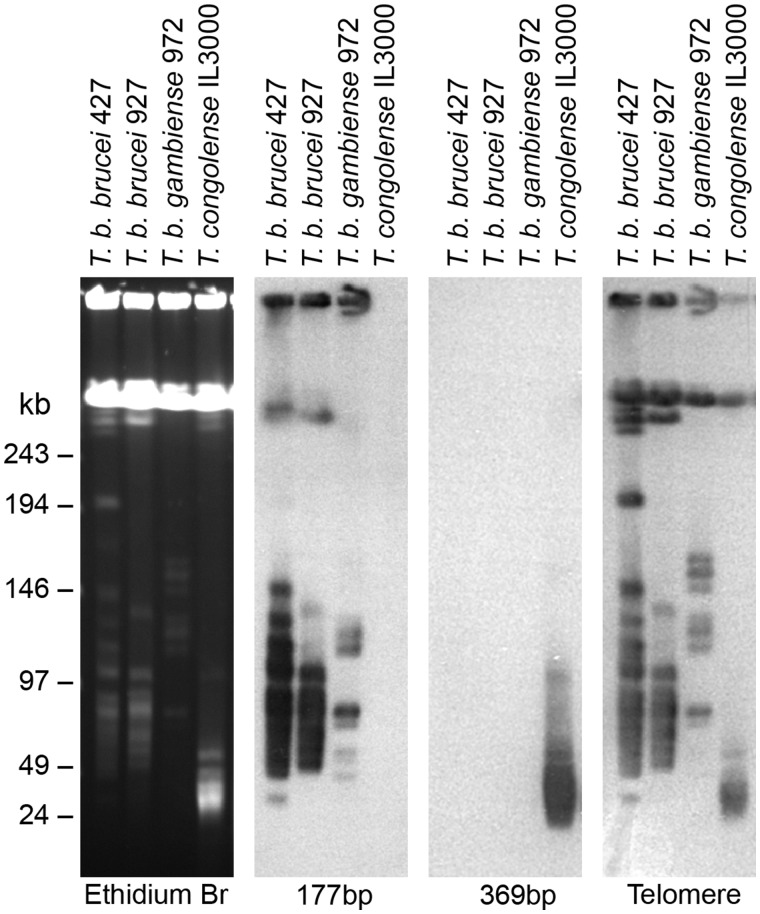
—Minichromosomal content of African trypanosome genomes. Genomic DNA of *T. brucei brucei* (strains Lister 427 and TREU927), *T. brucei gambiense* DAL972 and *T. congolense* IL3000 was separated by pulsed field gel electrophoresis and stained with ethidium bromide (first panel, reading left to right), hybridized to the *T. brucei* 177 bp repeat sequence (second panel), the *T. congolense* 369 bp repeat sequence (third panel), and telomeric repeats (fourth panel).

Note that when stating properties of the telomeric regions, we will give values for each strain thus: (Tc1/148/TcIL3000). Early studies of the *T. congolense* karyotype described minichromosomes containing a 369 bp satellite repeat, analogous to the 177 bp repeat of *T. brucei* (Gibson et al. 1988; Moser et al. 1989). Our data show that the 369 bp repeat hybridizes exclusively to DNA of minichromosomal size in *T. congolense* (fig. 2). The minichromosomes of *T. congolense* are both smaller (20–100 kb, with the majority between 20 and 60 kb) and more numerous than those of *T. brucei*. Estimates from the integrated intensity of ethidium binding to minichromosomes against single-copy chromosomes (as previously performed for *T. brucei;*Cross et al. 2014) indicate that minichromosomes represent 7–10 Mb of DNA in *T. congolense*, which for an average minichromosome size of ∼30 kb suggests there are 240–320 individual minichromosomes in a diploid *T. congolense* cell. We observed the 369 bp repeat in 80%/99% of telomere-containing contigs (see below), equating to ∼120 unique minichromosomal ends (∼20% of the total ends present, assuming that all minichromosomes possess the repeat). In complete minichromosome sequences, the 369 bp repeat forms a core up to 25 kb in length, with two telomeric regions at either end (fig. 1*a*). The length of this repeat appears to define the minichromosome size, as the region from the repeat to the telomere is only ∼5 kb in the majority of minichromosomal ends. Apart from a deletion of nucleotides 241–250 in strain Tc1/148, such that the repeat is 359 bp rather than 369 bp as in TcIL3000, the sequence and location of the 369 bp repeat is broadly conserved in both strains and between contigs. The sequence of the repeat is provided in supplementary fig. S1, Supplementary Material online.

**Fig. 3.**
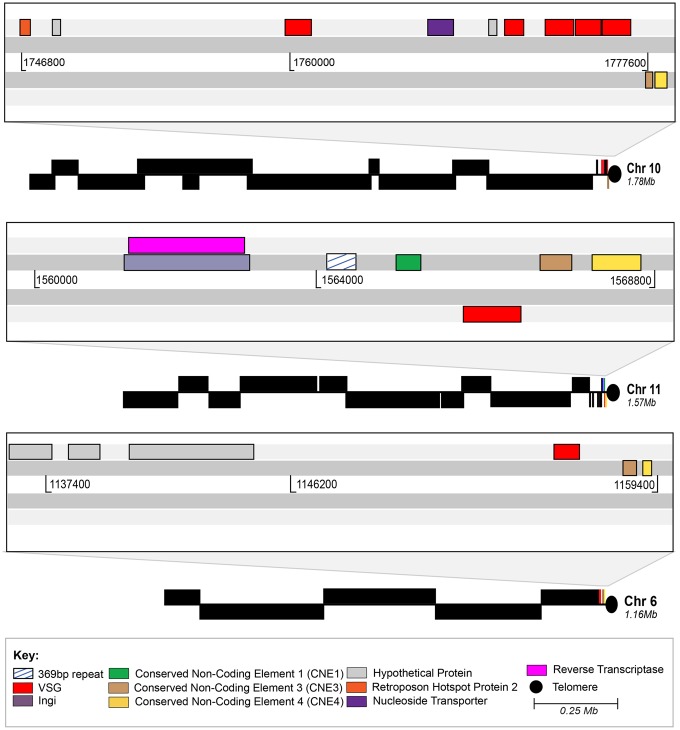
—Association of the conserved telomeric region with a megabase chromosomal assembly in Tc1/148. The cartoons show three instances of megabase-sized sequence assemblies that terminate with a telomeric repeat. These contigs correspond to chromosomes 10, 11, and 6 of *T. congolense* IL3000 by sequence homology. All chromosomes possess a telomeric VSG, but chromosome 11 (middle panel) also displays the complete, conserved telomeric region observed in figure 1; whereas chromosomes 6 and 10 only include CNE3 and 4 (lacking the core repeat and CNE1). Black boxes represent polycistrons displayed above and below the plane to reflect forward and reverse orientation respectively; black circles represent the telomeric repeat. Artemis representations of the sequences between the polycistrons and telomeres are shown in detail in the gray boxes above each contig. Coloured boxes represent conserved coding and noncoding features of the conserved telomeric region, defined in figure 1. Contigs are drawn to scale and have been aligned at their 3′ end. Conserved features are shaded according to key.

The minichromosomes of *T. congolense* have a structure similar to that seen for small chromosomes in *T. brucei* with a repetitive core region surrounded by variable subtelomeric DNA. For the assembled Tc1/148 *T. congolense* minichromosomes the 369 bp repeat region is uni-directional. However, the 177 bp repeat of *T. brucei* forms a large palindrome for at least some minichromosomes (Wickstead et al. 2004), as seen for 2 of 6 IL3000 minichromosomes (fig. 1*c*). Although Southern blotting demonstrated that most 369 bp repeats are contained in the minichromosomes (fig. 2), our assembly does contain one contig that places it adjacent to the telomere of a megabase chromosome, that is, chromosome 11 in Tc1/148 (*scf7180000002237*; fig. 3). The other 2 telomeric contigs that were scaffolded onto chromosomes 6 (*scf7180000002239*) and 10 (*scf7180000002595*), respectively, lacked the complete conserved telomeric region, but did contain a *VSG* locus and two CNEs (fig. 3).

**Fig. 4.**
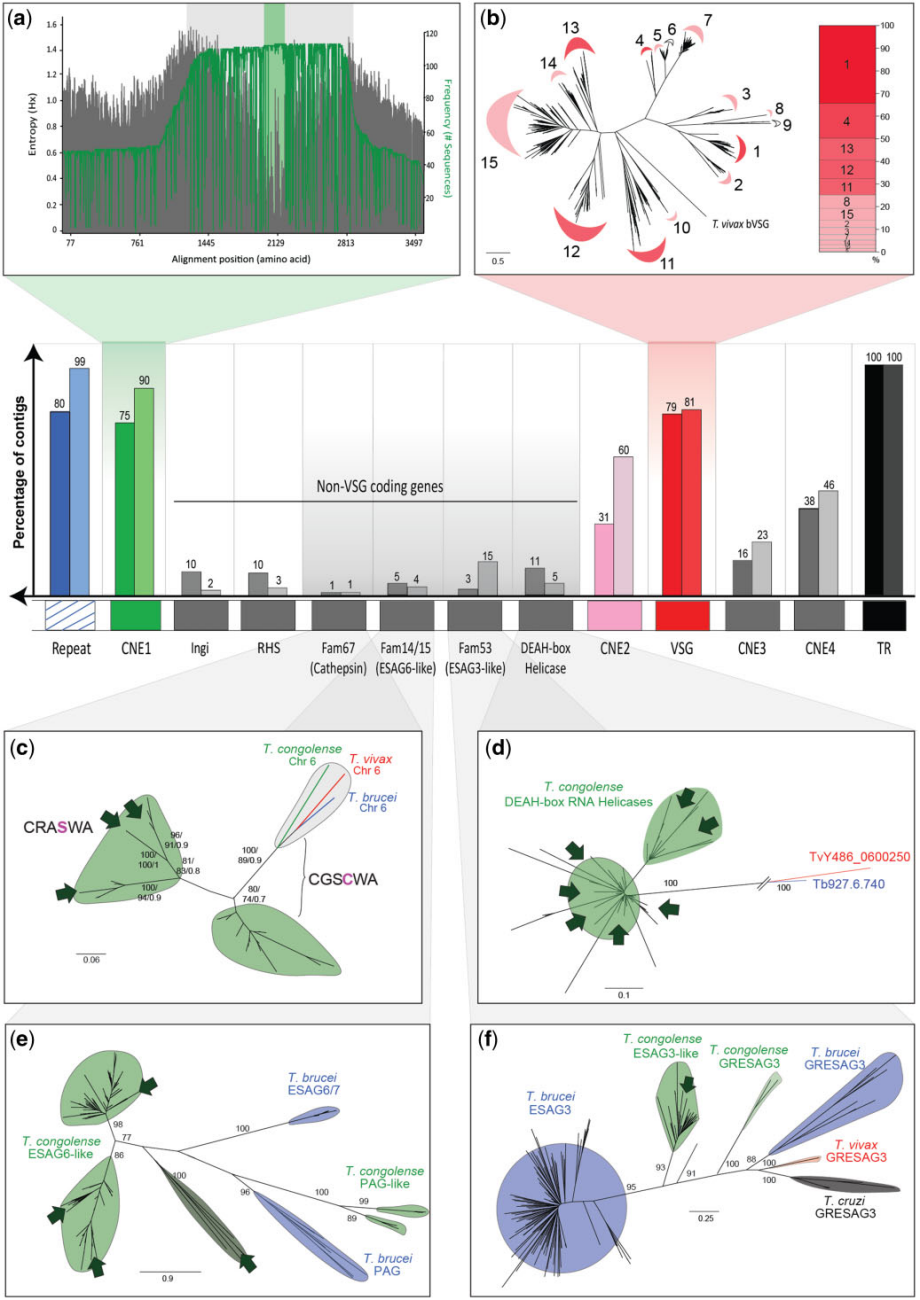
—A summary of gene content, order and variation across all conserved telomeric regions in *T. congolense* 1/148 and IL3000. The central panel shows the frequency of coding and noncoding sequence features observed within the conserved telomeric region, as a percentage of all telomere-containing contigs observed in 1/148 (left hand bars) and IL3000 (right hand bars). The displayed order of features reflects the observed orientation, with the 369 bp repeat region and conserved, noncoding element 1 (CNE1) found upstream of CNE2 (when present) and the *VSG*, and with CNE3 and 4 (when present) interposed between VSG and telomere. Non-VSG coding sequences are positioned as they were observed (i.e., between CNE1 and 2), but the precise order sequence of genes is only notional; in reality, non-VSG coding sequences were rarely observed and never all together in a single contig. (*a*) Sequence conservation around CNE1, expressed by Shannon entropy values for the multiple sequence alignment (left axis, in gray) and the residue conservation across contigs (right axis, in green). CNE1 corresponds most precisely with the low entropy-high conservation region shaded light green, and, to a lesser extent, with the wider region shaded light gray. (*b*) Unrooted Maximum Likelihood phylogeny of Tc1/148 *VSG* amino acid sequences estimated with PHYML (Guindon et al. 2010) with a WAG+Γ model. The contribution of phylotypes (labeled 1–15), to telomeric *VSG* repertoire (i.e., those VSG occupying the most telomere-proximal position within each contig) is shown in the cumulative bar chart to the right. (*c*) Unrooted Maximum Likelihood phylogeny of cathepsin B amino acid sequences (Fam67) estimated by PHYML with a WAG+Γ model and 100 bootstrap replicates. All Fam67 sequences from the Tc1/148 and IL3000 genome sequences are included, with an outgroup comprising a single-copy cathepsin-B gene from *T. congolense*, *T. brucei*, and *T. vivax*. Fam67 sequences associated with conserved telomeric regions in this study display a cysteine to serine amino acid replacement, previously observed in *T. congolense* (Mendoza-Palomares et al. 2008). (*d*) Unrooted Maximum Likelihood phylogeny of ATP-dependent DEAH RNA helicase amino acid sequences estimated with PHYML with a VT+Γ+F model and 100 bootstrap replicates. An outgroup of single-copy orthologs from *T. brucei* and *T. vivax* is included. (*e*) Unrooted Maximum Likelihood phylogeny of transferrin receptor-like amino acid sequences (Fam14/15) estimated with PHYML with a JTT+Γ model and 100 bootstrap replicates. Outgroups consist of PAG-like genes from *T. brucei* and *T. congolense*. (*f*) Unrooted Maximum Likelihood phylogeny of ESAG3-like amino acid sequences (Fam53) estimated with PHYML with a WAG+Γ model and 100 bootstrap replicates. Outgroups comprising low-copy number GRESAG3 genes in multiple species are included. In all panels, bootstrap proportions higher than 70% are shown at internal nodes. Dark green arrows indicate the positions of *T. congolense* genes found within conserved telomeric regions.

A summary of the features observed, and their topological order across all telomere-containing contigs, is shown in figure 4. We identified a GC-rich, Conserved Non-Coding Element (CNE1) in 75%/90% of contigs containing telomeric repeats (shaded green in figures). At its greatest extent, CNE1 is a 3,584 bp region containing no discernible protein-coding genes. Not all sequences share the entire region, however; a core of 1,368 bp with 65% sequence identity is common to all contigs (light gray shading in fig. 4*a*), and within this an ultraconserved core of 302 bp shows 87% sequence identity (light green shading in fig. 4*a* and supplementary fig. S2, Supplementary Material online). In complete minichromosomes, CNE1 is seen adjacent to both ends of the 369 bp repeat, suggesting that it is a regular part of the telomeric region repeated across the genome (fig. 1*a* and *b*). In most cases, CNE1 is in the same orientation as the *VSG*, where present (59%/60%).

We identified three further CNEs (CNE2–4) that are not found elsewhere in the genome by BLASTn searches, and so are specific to the telomeric region of both mini- and megabase chromosomes (figs. 1 and 3). Although CNE2–4 are not present in all instances (or even a majority of telomere-containing contigs), they are not mutually exclusive and they maintain their position relative to each other and to other features (see fig. 1 for multiple examples, and summary of all instances in fig. 4). CNE2 is the most abundant, composed of 180 bp repeats (supplementary fig. S3, Supplementary Material online) and found in 31%/60% of contigs, downstream of CNE1 and ∼2.5 kb upstream of a *VSG* gene. CNE3 and 4 are always located downstream of a *VSG* gene. CNE3 is a well-conserved and nonrepetitive 200 bp sequence (supplementary fig. S4, Supplementary Material online) found in 16%/23% of telomere-containing contigs. It is usually located ∼1 kb downstream of the *VSG* and ∼0.5 kb away from the telomere. CNE4 is a 150 bp element containing a 46 bp AT-rich motif (supplementary fig. S5, Supplementary Material online) present in 38%/46% of contigs; it is typically found ∼1.5 kb downstream of the *VSG* and ∼0.5 kb away from the telomere. In most instances (i.e., 81%/63% for CNE2, 56%/100% for CNE3, and 71%/96% for CNE4), these CNEs occur in a single orientation with respect to the telomere.

Although both CNE3 and CNE4 are annotated in the original IL3000 genome as coding sequences (i.e., TcIL3000_04880 and TcIL3000_0_12610), there are good reasons to consider them noncoding. First, there is no evidence for their expression among published *T. congolense* EST libraries of all life stages (Helm et al. 2009), or within an unpublished TcIL3000 bloodstream stage transcriptome (Wellcome Sanger Institute, https://www.ebi.ac.uk/arrayexpress/experiments/E-ERAD-440/). Our own epimastigote and metacyclic transcriptomes similarly contain no sequences derived from these loci (Silva Pereira et al. 2018). Second, they commonly have multiple internal stop codons. And third, they often have an elevated GC content (mean 60%) unlike the average for *T. congolense* coding sequences (51%). Another feature we note is that both CNE3 and 4 appear to be preferentially associated with *VSG* pseudogenes; in Tc1/148, 90% of *VSG* adjacent to CNE3 and 83% adjacent to CNE4 were pseudogenes, despite 62% of all telomeric *VSG* we observed having intact gene sequences.

### *Trypanosoma congolense* Telomeric Regions may Include Plausible VES

The most abundant, and often only, protein coding genes in the telomeric regions are *VSG*. *VSG* genes or pseudogenes are found in 79%/81% of telomere-containing contigs. In most contigs, they are the most telomere-proximal coding sequence (fig. 1), being positioned between 598 and 1,512 bp away from the telomeric repeat. As noted above, CNE3 and 4 are interposed between the *VSG* and telomere on occasion. This is the general situation depicted in figure 4; only in a few situations are *VSG* found further away from of the telomere. In these cases, there is normally multiple *VSG* within the contig, at least one of which is adjacent to the telomere. In *T. brucei*, ∼70% of *VSG* are either frame-shifted or fragmentary pseudogenes (Berriman 2005), and this includes many found adjacent to telomeres in expression sites (Hertz-Fowler et al. 2008). In both *T. congolense* strains, we find that most (62%/93%) *VSG* found in conserved telomeric regions are intact genes predicted to encode functional *VSG*, which is consistent with the much lower prevalence of pseudogenes across the genome compared with *VSG* in *T. brucei* (Jackson et al. 2012). Most of the 15 *T. congolense* VSG phylotypes (Jackson et al. 2012) are represented, although not proportionally to their genomic copy number (fig. 4*b*); for example, phylotype 15 is the most abundant phylotype in both strains (26%/17%) but a relatively minor contributor to telomeric loci (4%/9%). Conversely, phylotype 1 is a relatively minor component of genomic repertoire (7%/7%) but the largest contributor to Tc1/148 telomeric loci (35%). Two of the smallest phylotypes, 6 and 9, were not observed in any telomeric region in either Tc1/148 or IL3000.

We contend that similarities with the context of telomeric *VSG* gene expression in *T. brucei*, and the knowledge that *VSG* expression in *T. congolense* is associated with the telomere (Majiwa et al. 1985), indicate that these conserved telomeric regions either include a VES(s), or otherwise reflect the structure of the active VES.

### Protein Coding Genes Within *T. congolense* Telomeric Regions are Not Orthologous to *T. brucei* ESAGs

In *T. brucei* bloodstream-stage VES, ESAGs are regular features that encode diverse bloodstream-stage proteins with predominantly membrane functions (Hertz-Fowler et al. 2008; Jackson et al. 2013). We asked if *T. congolense* telomeric regions contain orthologs to *T. brucei* ESAGs, or else, different genes specifically associated with telomeric regions. Generally, we found that non-*VSG* genes were infrequent, being present in 19%/22% of telomeric contigs. Among these genes were features normally associated with African trypanosome subtelomeres such as the transposable element *ingi* and retrotransposon hot spot (RHS) protein pseudogenes, which occurred in 10%/3% of telomeric regions (fig. 4). Six additional coding sequences representing four hypothetical proteins were found in the context of the Tc1/148 telomeres, corresponding to genes from the original IL3000 assembly: TcIL3000_0_16860 (*scf7180000002509*), TcIL3000_0_59850 (*scf7180000002475*), TcIL3000_0_02720 (*scf7180000002537*), and TcIL3000_0_51940 (*scf7180000002675*). Figure 4*c*–*f* describes the phylogenies of four multicopy gene families that we observed in the telomeric context multiple times: Cathepsin-B cysteine protease (or Fam67; fig. 4*c*), DEAH-box RNA helicases (fig. 2*d*), transferrin receptor genes (or Fam15; fig. 4*e*), and ESAG3-like genes (or Fam53; fig. 2*f*). Although these were the most common non-*VSG* genes observed, it should be noted that they were infrequent nonetheless, never occurring in more than 15 cases.

In *T. brucei* and *T. vivax*, cathepsin B is a single-copy gene but it is known to have duplicated and diversified in *T. congolense*, being present at multiple subtelomeric loci in addition to the conserved locus on chromosome 6 (Mendoza-Palomares et al. 2008). These genes were shown to be essential for *T. congolense* survival, and were implicated in lysosomal protein degradation and immunogenicity. Furthermore, some of the species-specific cathepsin-B genes contain a cysteine to serine amino acid replacement at position 369, which affects the known catalytic site (Mendoza-Palomares et al. 2008). We found two contigs in Tc1/148 (contigs *scf7180000002509* and *scf7180000002621*) and one contig in IL3000 (contig *PQVL01000138*.1) containing cathepsin-B, all displaying the serine residue in the catalytic site. In addition to these, 8/17 cathepsin B gene sequences were recovered from subtelomeric loci. In a phylogeny, cathepsin-B genes associated with the telomeric region do not form a separate clade, but are paraphyletic with subtelomeric homologs, suggesting repeated transposition of these genes between the two genomic domains (fig. 4*c*).

DEAH-box RNA helicase genes were found in 11%/5% of the telomeric regions, occasionally arranged in tandem pairs. These genes were most similar to TcIL3000_6_260, a single-copy gene located at a strand-switch region of chromosome 6 and conserved in all trypanosomatids. Like cathepsin-B, our phylogenetic analysis shows that this single-copy gene has been uniquely duplicated in *T. congolense*; we found 24/17 copies distributed among subtelomeric loci. Again, like cathepsin-B, the genes we identified in telomeric regions were not monophyletic, or otherwise distinct from subtelomeric homologs (fig. 4*d*); they seem to reflect a genome-wide gene family elaboration, rather than a feature of telomeric regions per se.

The heterodimeric transferrin-receptor is encoded by ESAG6 and 7 in *T. brucei*, and these genes are arranged in tandem pairs within the bloodstream VES (Salmon et al. 1994). The *T. congolense* IL3000 genome contains multiple homologs, which are co-orthologous to ESAG6 (Jackson et al. 2012, 2013). Collectively, these transferrin receptor genes are referred to as Fam15 (Jackson et al. 2013). Although in *T. brucei*, Fam15 genes are almost exclusively found within the VES, in *T. congolense* Fam15 genes are distributed throughout the subtelomeres, often in tandem pairs (Jackson et al. 2013). In contrast, Fam15 genes were found in only 5%/4% of our telomeric regions (fig. 4), and never as tandem pairs. Two instances can be seen in figure 1*b* where a single Fam15 gene (light blue) is positioned adjacent to the telomere of a minichromosome (contigs *scf7180000002408* and *scf7180000002549*). The remaining telomeric copies (*N* = 5 in both strains) were found upstream of the *VSG* gene. Phylogenetic analysis shows that the *T. congolense* transferrin receptors found in telomeric regions are not monophyletic, and are indistinguishable from subtelomeric versions in 1/148 and IL3000 (*N* = 86/50, respectively) (fig. 4*e*). Therefore, *T. congolense* transferrin receptor genes found within the telomeric region were not orthologs to ESAG6/7, confirming a conclusion based on subtelomeric copies (Jackson et al. 2012).

Fam53 encodes ESAG3 and ESAG3-like proteins in all trypanosomatids (Jackson et al. 2012). In *T. brucei*, Fam53 is a large gene family consisting of ESAG3, located both within the bloodstream VES and throughout subtelomeric loci (most copies of which are pseudogenes), as well as another clade of less numerous, and more divergent, “GRESAG3” sequences. The latter represent an ancestral lineage, which is conserved in other trypanosomatids, whereas ESAG3 *sensu stricto* was said to be a *T. brucei*-specific expansion (Jackson et al. 2012). In this study, Fam53-like genes were found in 3%/15% of telomeric regions, and these sequences are distinct from *T. congolense* GRESAG3. Eight of the 11 copies of this novel lineage were also found at subtelomeric loci. Thus, the situation in *T. congolense* seems to replicate that in *T. brucei*. Figure 4*f* shows how, like *T. brucei*, *T. congolense* has evolved an expanded Fam53 repertoire, distinct from the ancestral GRESAG3 clade, which is largely located in the subtelomeres. As in previous cases above, the paraphyly of Fam53 genes from conserved telomeric regions suggests that these genes routinely transpose between subtelomeric and telomeric loci.

Therefore, although non-*VSG* genes occur within *T. congolense* telomeric regions, they are either nonhomologous to *T. brucei* ESAGs, or paralogous. Furthermore, none of the would-be *T. congolense* “ESAGs” display the same unique association with the telomeric context that is evident in *T. brucei*, as they are not structurally distinct from paralogs found at numerous subtelomeric loci.

### Recombination is a Driver of Sequence Evolution in *T. congolense* Telomeric Regions

**Fig. 5.**
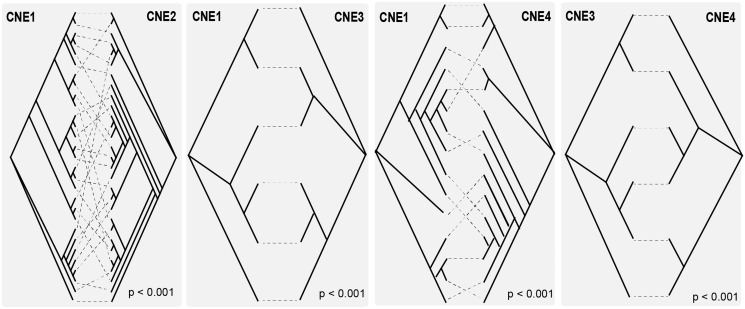
—Topological differences between the phylogenetic relationships of CNEs (CNE1–4) when found within the same conserved telomeric regions. Each of four tanglegrams shows a pair of phylogenies describing the relationships of two CNEs from the same set of telomere-containing contigs (see supplementary file S1, Supplementary Material online for accession codes). Dashed lines link CNEs from the same contig. In the absence of structural reorganization, the branching pattern of CNEs should be identical. In all cases, the topologies are significantly different according to a Shimodaira–Hasegawa test (Shimodaira and Hasegawa 1999) conducted in RaxML (Stamatakis 2014).

**Fig. 6.**
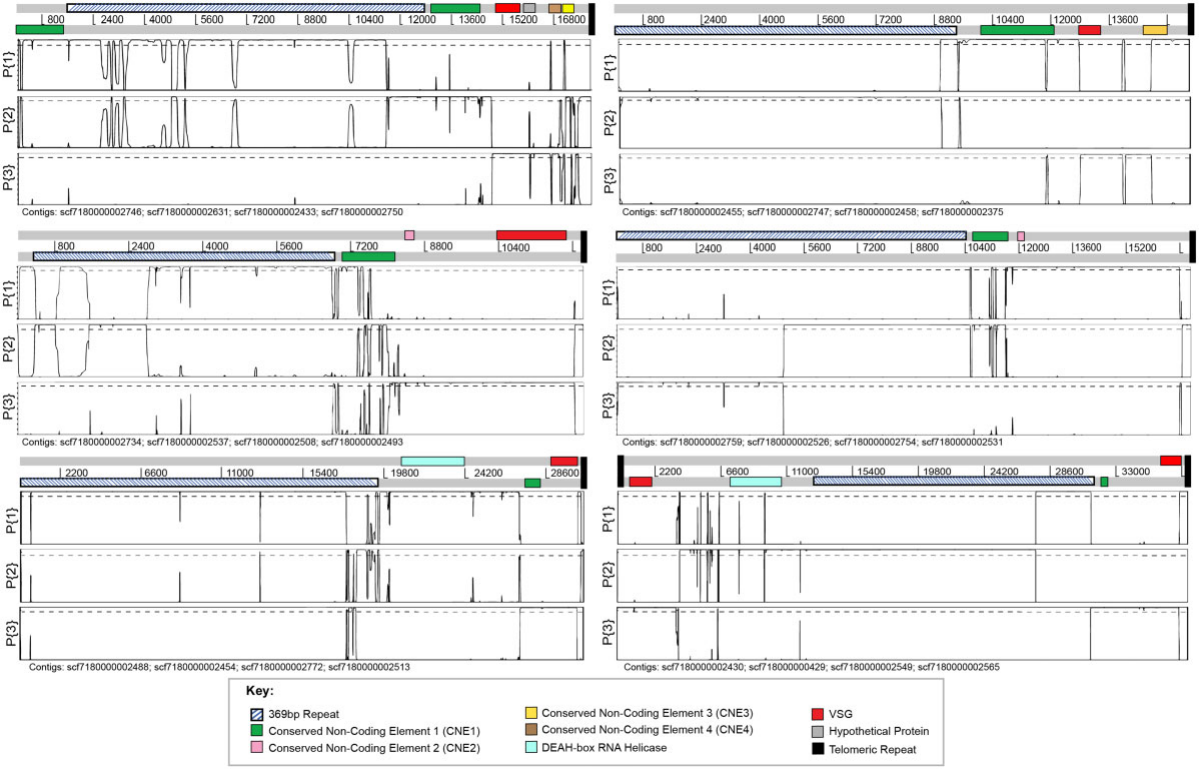
—Phylogenetic incompatibility within sequence alignments of conserved telomeric regions. Each panel describes the phylogenetic signal detected in a multiple sequence alignment of four telomeric regions containing similar features (six out of 16 total comparisons). At the top of each panel, the features of one sequence in the quartet are shown as in Artemis; conserved features are colored according to key. Beneath this, three panels plot the Bayesian probability of each possible phylogenetic topology (P{1–3}) at positions along the alignment, measured with Topali (Milne et al. 2009). When a black line moves above the significance threshold (*P* = 0.95) given by the dashed line, the corresponding topology becomes optimal. Changes in optimality indicate recombination breakpoints. The contigs used in each sequence quartet are named below each panel.

Recombination is thought to play a frequent and crucial role in regulating VES sequences in *T. brucei*. To assess whether *T. congolense* telomeric regions are reorganized through homologous recombination, we analyzed the differences in phylogenetic signal across conserved features. In the absence of recombination, the phylogenetic relationships between features along the canonical structure should be consistent, only reflecting the pattern of telomere duplication. However, if homologous recombination plays a role in sequence evolution, the relationships among telomeric regions will change depending on which marker within the sequence is considered. Figure 5 shows that there are significant topological differences between phylogenies for CNE1 and CNE2–4 derived from the same contigs, and also between CNE3 and CNE4 trees. Thus, there is no consistent evolutionary history for all sequences, which is consistent with positions becoming decoupled due to recombination. To examine this further, Phi (Bruen et al. 2006) and Topali (Milne et al. 2009) were used to further characterize recombination breakpoints along the telomeric regions. A total of 16 groups of 4 contigs containing at least two conserved features were evaluated. Phi found evidence for recombination in 14 of 16 quartets (87.5%). Figure 6 shows putative recombination breakpoints in six of these alignments detected by Topali, which calculates the Bayesian probability of the three possible phylogenetic topologies along the alignment, and identifies the points where the most probable topology changes. Switches in phylogenetic signal occur most often between the 369 bp repeat and CNE1, and between CNE1 and the *VSG*. These results suggest that *T. congolense* telomeric regions are reorganized through frequent recombination.

## Discussion

Improved genome assemblies based on long PacBio sequences have allowed us to describe contigs from *T. congolense* that indicate unprecedented numbers of telomeres with respect to trypanosomatid genomes. Our analysis of 281 such contigs from two *T. congolense* strains has identified a conserved telomeric region associated with telomeres of both minichromosomes and perhaps also larger chromosomes. At its most basic, this region consists of a 369 bp-repeat sequence, a conserved, noncoding element (CNE1) and, in approximately half of cases, a telomeric *VSG* gene.

The first of these conserved features, the 369 bp repeat, was proposed as a minichromosomal marker when first observed in *T. congolense* (Kukla et al. 1987; Gibson et al. 1988; Moser et al. 1989). The *T. congolense* and *T. brucei* genomes include numerous minichromosomes containing AT-rich and species-specific repeats (Williams et al. 1982; Sloof, Menke, et al. 1983). The 369 bp repeat was thought to be analogous to the 177 bp repeat in *T. brucei* (Sloof, Bos, et al. 1983; Gibson et al. 1988), and similar motifs in *T. vivax* (170 bp; Dickin and Gibson 1989). We have resolved the complete structure of a number of minichromosomes in *T. congolense*. The overall architecture for these chromosomes is similar to *T. brucei:* a core of tandem repeats, flanked by telomeric *VSG* loci. In *T. brucei* Lister 427, the mean minichromosomal size is ∼75 kb, and there are approximately 100 minichromosomes per cell (Cross et al. 2014). We show that *T. congolense* minichromosomes are smaller (most between 20 and 60 kb), but much more numerous (∼300 per cell). In both species, these repeats appear to delimit the chromosome-internal boundary of a conserved, peri-telomeric region, and perhaps offer a focus for homologous recombination and telomeric exchange.

The second conserved feature of *T. congolense* telomeric regions is a noncoding element immediately downstream of the 369 bp repeat. The high sequence conservation of CNE1 suggests that it may have an important regulatory function. One possibility is that it contains a promoter for the putative VES. Some features of CNE1 support this role: It is located in a similar position relative to the 369 bp repeat as the *T. brucei* VES promoter is relative to the 50 bp repeat; it is present in 75%/90% of instances and is always positioned upstream of any gene loci, including the *VSG* if present; and it contains no plausible protein coding sequences. Furthermore, a core 302 bp region within CNE1 displays 87% sequence identity across all instances, indicating either strong functional constraints or very frequent recombination among multiple sites. CNE1 is not always positioned in the same orientation as the telomeric *VSG*, however, in *T. brucei*, sequence reorganization can produce silent VES lacking promoters, or with promoters in atypical positions (Zomerdijk et al. 1990; Gottesdiener et al. 1991; Horn and Cross 1997), so perhaps active transcription only occurs where orientation is coordinated. If CNE1 is a promoter, this suggests that *T. congolense VSG* could be expressed directly from minichromosomes, as has been suggested before (Majiwa et al. 1986). This is not seen in *T. brucei*, where minichromosomes typically lack promoters and are transcriptionally silent (Sloof, Bos, et al. 1983; Wickstead et al. 2004). To be expressed in *T. brucei*, a minichromosome *VSG* must be transposed into a VES that are, as yet, only found on megabase- or intermediate-sized chromosomes (Cross et al. 2014). This could mean that *T. congolense* has a larger repertoire of potential expression sites than is available in *T. brucei*. Alternatively, CNE1 may have another regulatory function, such as being the source of a long noncoding RNA, or providing a binding site for proteins regulating *VSG* expression.

The combination of the features described above with a telomeric VSG locus in 80% of telomeric regions, coupled with the general similarity to the *T. brucei* VES, suggests to us that the *T. congolense* VES has this topology. Admittedly, most of the telomeric regions we describe are attached to minichromosomes and, if *T. congolense* is like *T. brucei* and *VSG* are expressed from larger chromosomes only, this may mean that an active VES is not among our sequences. However, we have found the same combination of 369 bp repeat, CNE1 and *VSG* to be associated with chromosome 11 (*scf7180000002237*) in one contig, suggesting the same telomeric context may occur on mini- and (some) megabase chromosomes. Hence, regardless of whether *T. congolense VSG* expression originates from mini- or megabase chromosomes, we suggest that it will be associated with the telomeric structure we describe.

Sequence features of the *T. brucei* VES, such as the 70 bp repeat upstream of the *VSG* and the *VSG* C-terminal domain (CTD), are known to facilitate *VSG* transposition, leading to antigenic switching (Hovel-Miner et al. 2016). VSG transposition has been observed in *T. congolense* (Majiwa et al. 1985) and the phylogenetic incompatibility we see within alignments of telomeric regions is indirect evidence of regular antigenic switching, comparable to the patterns seen in *T. brucei* Lister 427 (Becker 2004; Hertz-Fowler et al. 2008). On multiple occasions, *VSG* genes and their flanking CNE3/4 present a different phylogeny to CNE1 and upstream elements, indicating a recombination breakpoint upstream of the *VSG*. Yet, the 70 bp repeat and the universal CTD do not exist in *T. congolense*, whose VSG have diverse CTD, conserved within phylotypes (Jackson et al. 2012). *VSG* CTD could act as 3′ anchor points for homologous recombination, but this would only facilitate recombination between members of the same *VSG* clade, with homologous CTD. The corresponding 5′ annealing point is unclear; we see nothing comparable to the 70 bp repeat. We have observed additional conserved, noncoding features (CNE2–4) around the *VSG* locus that could be involved in sequence transposition, either as 3′ or 5′ annealing points. However, they are present in only a small minority of cases, and not beyond the conserved telomeric regions. Thus, they might facilitate ectopic gene conversion between telomeric regions, but not recruitment of *VSG* from subtelomeric loci.

If CNE2–4 are not involved in VES sequence reorganization, they may regulate VES activity. Noncoding elements have regulatory roles in other antigenic variation systems. For example, in the malaria parasite *Plasmodium falciparum*, expression of long-coding RNA from within the *var* gene locus is required for *var* gene activation (Jiang et al. 2013). Furthermore, conserved noncoding DNA elements, such as the 5′ UTR, have been associated to *var* gene activation and silencing (reviewed by Frank and Deitsch 2006). Similarly, in *Babesia bovis*, the cause of bovine babesiasis, expression of the variant erythrocyte surface antigen-1 (VESA1) is controlled by a bidirectional transcriptional promoter and multiple, noncoding regulatory elements flanking the *ves1* genes (Al-Khedery and Allred 2006; Wang et al. 2012). In *Babesia*, these noncoding elements both regulate promoter activation and drive in situ transcriptional switching (Wang et al. 2012). Thus, it could be that CNE2–4 encode ncRNA, or are binding sites for proteins involved in VES silencing or activation. Alternatively, CNE2–4 may not be adaptive at all, resulting instead from recent duplications of specific telomeric regions by biased gene conversion, resulting in several copies. Ultimately, only by observing the dynamic reorganization of the telomeric regions over time and during infections will we understand any potential involvement of CNE2–4 in antigenic switching.

A prominent feature of bloodstream-stage VES in *T. brucei* is the presence of ESAG1–13, in a well conserved gene order (Hertz-Fowler et al. 2008; Young et al. 2008). Previously, phylogenetic analysis showed that ESAGs had eclectic origins and most (i.e., ESAG2, 4–9 and 11) have derived sequences unique to the VES, distinct from their progenitor loci on the core chromosomes (Jackson et al. 2012). This study now confirms that *T. brucei* ESAGs are species-specific and no orthologs exist in the telomeric regions of *T. congolense* (although see below). Homologs to ESAG1, 5, 8, and 9–13 were never seen. Homologs to ESAG4 and ESAG6/7 were seen, but very sporadically. Furthermore, on the single occasion that an ESAG4 homolog located near the telomere, this sequence was more closely related to core chromosomal adenylate cyclase genes (i.e., “GRESAG4”) than ESAG4 itself. On a few occasions, homologs to ESAG6/7 (i.e., transferrin receptor genes) were seen, but these were unpaired and are much more numerous throughout the subtelomeres (unlike *T. brucei*). Although it is plausible that transferrin receptor genes might be activated through transposition into the VES, this seems unlikely because the telomere-associated genes we observed are not paired, unlike many of the subtelomeric loci (Jackson et al. 2012), and the functional transferrin receptor is heterodimeric (Salmon et al. 1994). The only plausible case of orthology between *T. brucei* and *T. congolense* concerns ESAG3. In both species, these ESAG3 sequences are distinct from “GRESAG3” sequences found in the chromosomal cores, which are orthologous with genes in other trypanosomatids. Therefore, the simplest explanation is that both *T. brucei* and *T. congolense* have inherited an expansion of ESAG3 sequences that transpose between subtelomeric and telomeric regions.

If an ESAG is simply any non-*VSG* gene within an expression site, *T. congolense* may have recruited a different set of genes into its own VES, but our analyses seem to largely discount this. We do observe additional coding sequences within telomeric regions (i.e., DEAH RNA helicase, cathepsin and RHS), however, they are not specific to the telomeric context. Both cathepsin and DEAH-box RNA helicase genes belong to *T. congolense*-specific expansions of otherwise single-copy genes found in all trypanosomatids; the telomere-associated genes are not monophyletic, nor structurally distinct from paralogs found in subtelomeric loci (fig. 4*c* and *d*). This indicates a greater degree of molecular adaptation of the VES in *T. brucei* for developmental regulation of diverse, bloodstream-stage genes besides *VSG*. It appears that *T. congolense* has not recruited genes to its telomeric regions in the same way as *T. brucei*, which may have important implications for their phenotypic differences while in the blood.

## Conclusion

We have reported a conserved peri-telomeric region associated with *T. congolense VSG* loci, largely associated with minichromosomes. Several similarities with the *T. brucei* VES exist. An intact, telomeric *VSG* gene is often present. A repeat sequence occurs distally from the telomere, delimiting the conserved region at the 5′ end. A highly conserved, noncoding sequence occurs upstream of the *VSG* locus, and may be a transcriptional promoter. Finally, the sequences of these telomeric regions appear to be affected by frequent recombination, and CNEs may facilitate this. However, in other features, the *T. congolense* sequences are distinct from *T. brucei* VES. The *VSG* loci are not flanked by conserved repeats that offer an immediate mechanism for antigenic switching. Would-be *T. congolense* ESAGs are relatively rare and not orthologous to *T. brucei* ESAGs (except possibly ESAG3). Moreover, where they occur, they are not derived forms uniquely associated with the telomeric context, but members of widespread gene families. Although this structure has little strict homology with the *T. brucei* VES, the obvious analogy suggests functional correspondence. It seems likely that the ancestor of *T. brucei* and *T. congolense* possessed a telomeric VES, but the daughter species have diverged in ways that may have important effects on their capacity for antigenic variation.

## Supplementary Material

Supplementary data are available at *Genome Biology and Evolution* online. 

## Supplementary Material

Supplementary Data
