# Supplementary material for: The Structure of a Conserved Telomeric Region Associated with Variant Antigen Loci in the Blood Parasite Trypanosoma congolense
Source: Genome Biol Evol. 2018 Aug 25;10(9):2458–73. doi: 10.1093/gbe/evy186 (PMC6152948; doi:10.1093/gbe/evy186)
Supplement: Supplementary Data [file evy186_supp.zip › Supplementary_Figures.pdf]

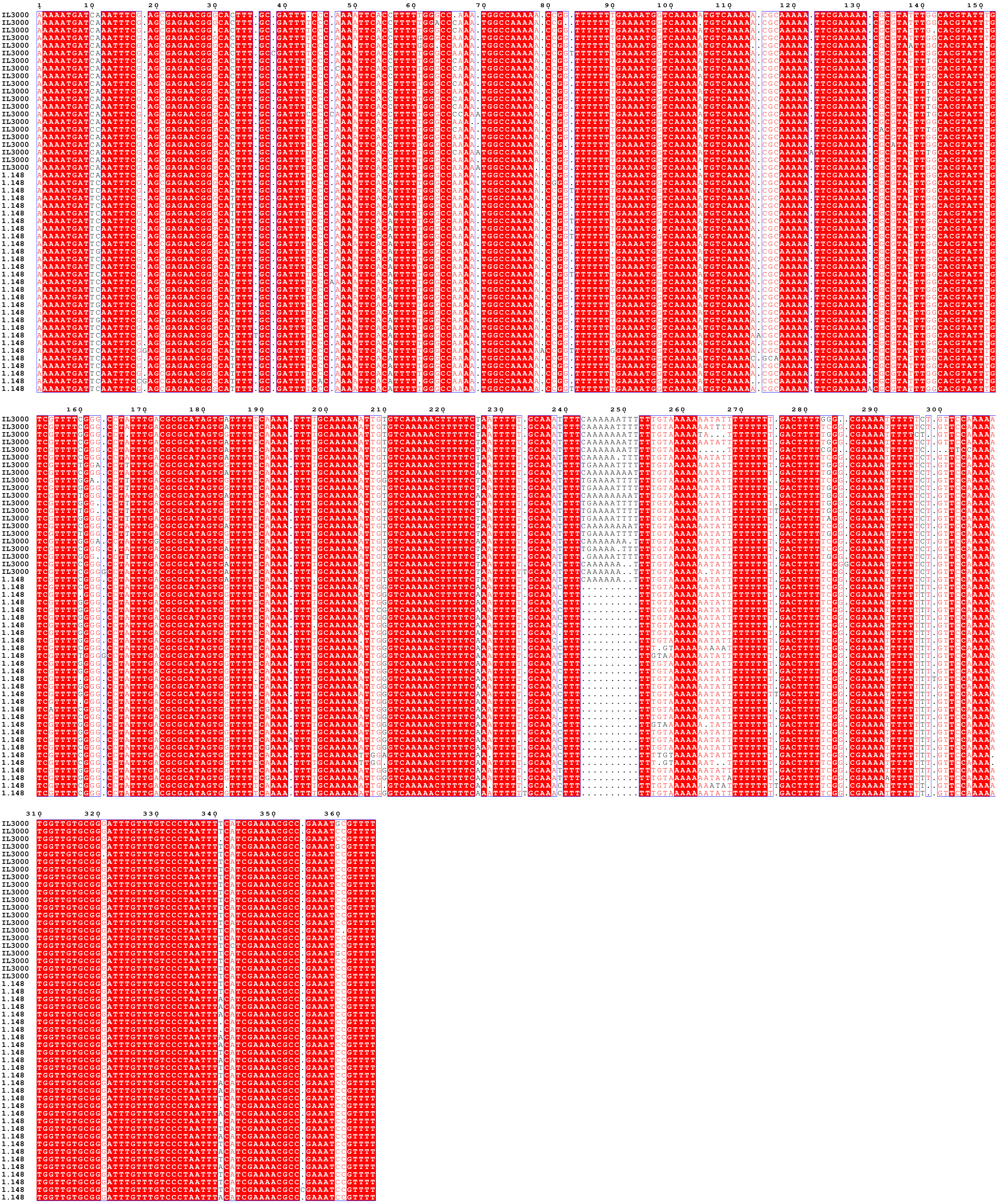

Supplementary Fig. 1 Multiple Sequence Alignment of the 369bp repeat in IL3000 and Tc1/148. Nucleotides shaded red have ≥70% identity across the alignment. For clarity, only 50 sequences are shown. Note the 10-nucleotide deletion in Tc1/148 around position 250 of the alignment. Image was produced with ESPript 3.0 (Robert, X. and Gouet,P. 2014).

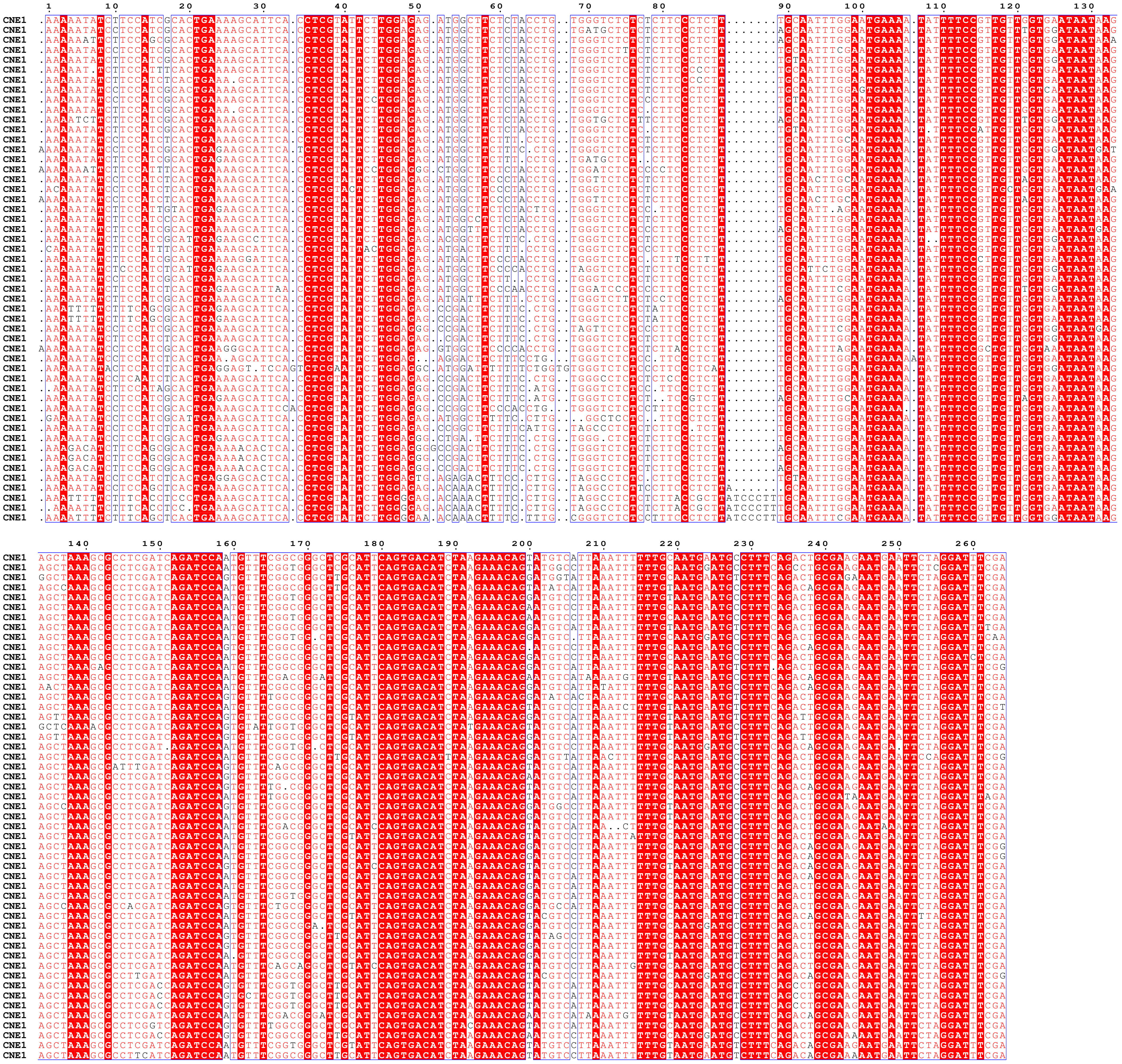

Supplementary Fig. 2 Multiple Sequence Alignment of the conserved core of CNE1. Nucleotides shaded red have ≥70% identity across the alignment. For clarity, only 50 sequences are shown. Image was produced with ESPript 3.0 (Robert, X. and Gouet,P. 2014).

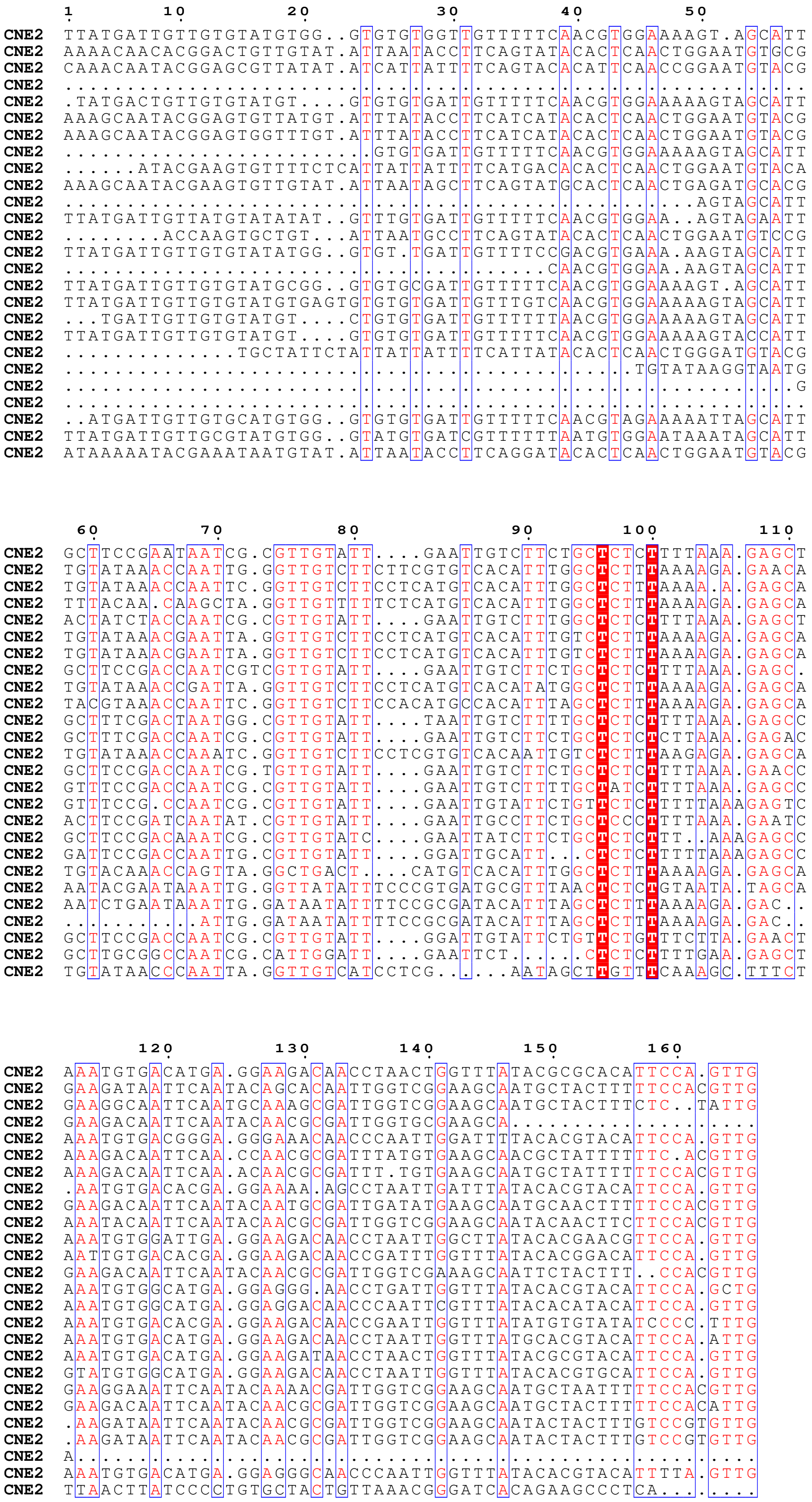

Supplementary Fig. 3 Multiple Sequence Alignment of CNE2. Nucleotides shaded red have ≥70% identity across the alignment. Image was produced with ESPript 3.0 (Robert, X. and Gouet,P. 2014).

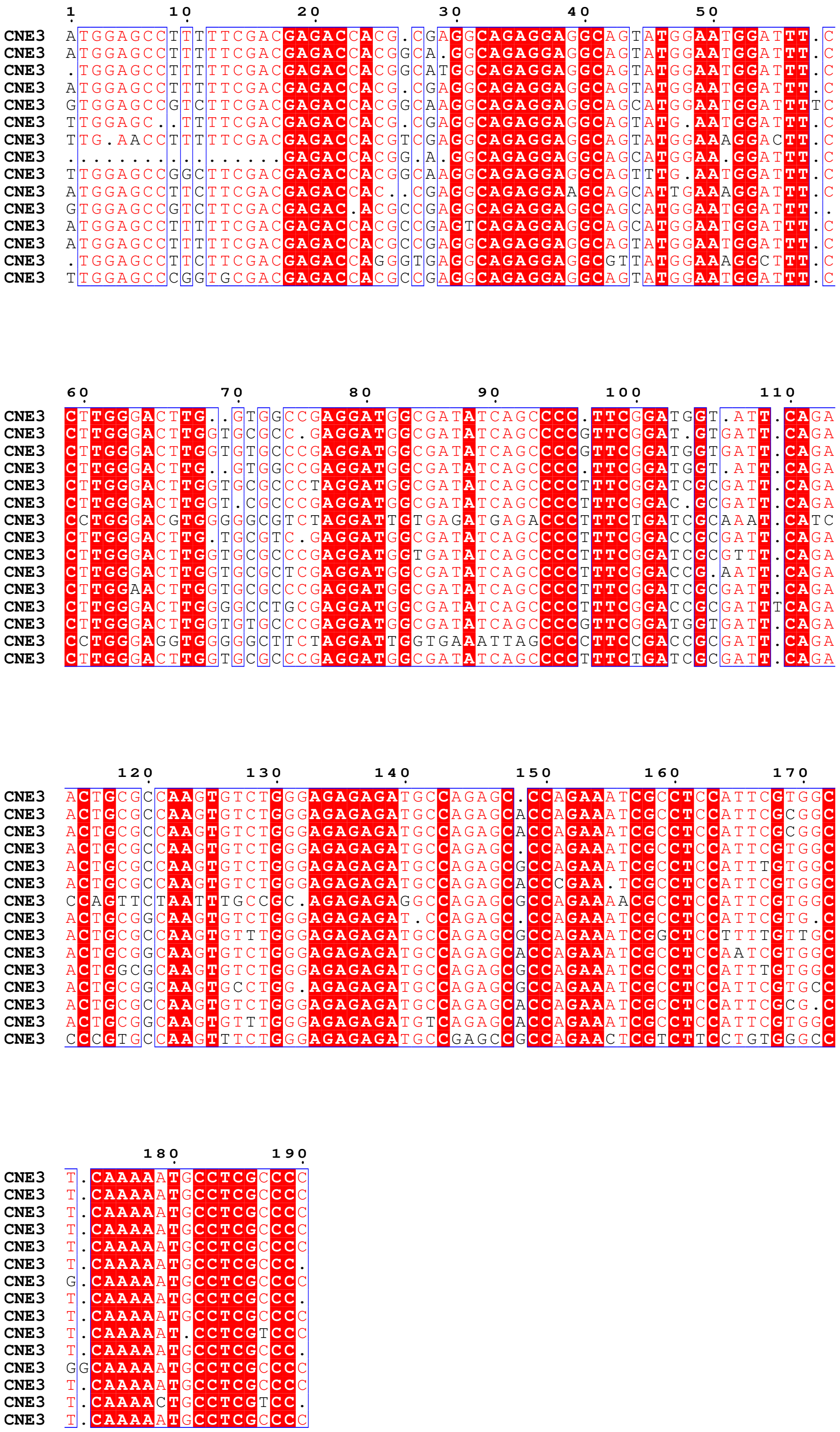

Supplementary Fig. 4 Multiple Sequence Alignment of CNE3. Nucleotides shaded red have ≥70% identity across the alignment. Image was produced with ESPript 3.0 (Robert, X. and Gouet,P. 2014).

```
          1        10        20        30        40        50
CNE4  ATGGGCGGGGGCAGGCAT.CG.AACTAAC.TTCGGAGTGGC..CTCCATCCAATGGC.GC
CNE4  CTGGGG.CGGGAAG.GCATCG.AAATAAC..TCGGAGTGGC..CTCCATCAACTGGCGCA
CNE4  CTGGGG.CGGGAAG.GCATCG.AAATAAC..TCGGAGTGGC..CTCCATCAACTGGCGCA
CNE4  CTGG...CGGGACAGGCATCG.AAATAAC.CACGGAGTGGC..CTCCATCAAATGGCGCA
CNE4  CTTGACGAGCCAGGACAGGTG.CGAGCGC.GTATCTGTGCG..CTACCACAAACGGC.AT
CNE4  CTGGACGACTCAGGACAGGTG.CGAGCGC.GTATCCGTGAG..CTACGACAAACGAC.TT
CNE4  CTGGACGACTCAGGACAGGT..CGAACGC.GTATCTGTGCG..CTACGACAAACGAC.TT
CNE4  CTGGACGAATCAGGACAGGTG.CGAGCGC.GTATCTGTGAG..CTACGACAAACGAC.TT
CNE4  CTGGAGGACTCAGGACAGGTG.CGAGCGC.GTATCTGTGCG..CTACGACGTACGAC.TT
CNE4  CTGGAGGACTCAGGGCAGGTG.CGAGCGC.GTATCTGTGCG..CTACGACAAACGAC.TT
CNE4  CTGGGCCACTCAGGACAGGTG.CGAACGC.GTATCCTTGAG..CTACGGCAAATGGC.TT
CNE4  CTGGCTAAAGATGGCTGGACAACTGGCACCGGCGCTATCAGTTCCACCTGGAATGCCCTC
CNE4  CTGGGGGCGGGAAGAGCATCG.AAATAAC.CTCGGAGTGGC..CTCCATCAACTGGCGCA
CNE4  CTGGG..CGGGGGAGGCATCG.AAATAGC.CACGGAGTGGC..CTCCATCAAATGGCGCC
CNE4  CTGGG..CGGAACCGGCATCG.AAATAAC.AACG.AATGGC..CTCCATCAAGTGGCGCA
CNE4  CTGGACGAATCAGGACAGGTG.CGAGCGC.GTATCTGTGAG..CTACGACAAACGAC.TT
CNE4  CTGGGCCACTCAGGACAGGTG.CGAACGC.GTATCCTTGAG..CTACGGCAAATGGC.TT
CNE4  CTGGAGGACTCAGGACAGGTG.CGAGCGC.GTATCTGTGCG..CTACGACGTACGAC.TT
CNE4  CTGGACGAC.CAGGACAGGC..GAAGCGC.GTATCTGTGAG..CTACGACAAACGGC.TT
CNE4  CTGGGC.GGGGACAGGCATCG.AAATAAC.CAAGGAATGGC..ATCCATCCAATGGCGCA

              60        70        80        90       100       110
CNE4  AAGACGAGCAGAGAATATCCTCCGTTGTGGAAAGCCTTAGAGTGGTCAATGGAACCATCG
CNE4  GAACCACAC.GAGA.TATCCTCCGTTGCGGAAAGCCTTAGAGTGGTAAATGAAACCATCG
CNE4  GAACCACAC.GAGA.TATCCTCCGTTGCGGAAAGCCTTAGAGTGGTAAATGAAACCATCG
CNE4  GA..CACAC.GAGAATATCCTCCGTTGCGGAAAGCCTTAGAGTGGTAAATGGAACCATCG
CNE4  GAAGCGAGCCGAGAAACTCCTCAGTTGCGGAAAGTCTTGGAGTGGTAAATAGAACCATCG
CNE4  GAATCGAGCCGAGAAACTCCTCAGTTGTGGAGAGCCTTAGAGTGGTAAATGGAACCGCCG
CNE4  GAATCGAGCCGAGAATCTC.TCAGTTGCGGAGAGCCATAGAGTGGTAAATGGAACCATCG
CNE4  GAAACGAGCCGAGAATATCCTCAGTTGTGGAAAGCCCTAGAGTGGTAAATGGGACCATCG
CNE4  GAATCAAGCCGAGAAACTCCTCAGCTGTGGAAAGCCTTAGAGTGCTAAATGGAACCATCG
CNE4  GAATCGAGCCGAGAATATCCTCATTTGTGGAGAGCCTTAGAGTGGTAAATGGAACCGCCG
CNE4  GAATCGAGCCGAGAATGTCGTCAGTTGTGGAGAGCCTTAGAGTGGTAAATGGAACTATCG
CNE4  ACCATGACCACAGGAGGCC..CAGTT.TGAAGATTATCACGGCCGACAATTTAGCCCCCT
CNE4  GAACCACAC.GAGAATATCCTCCGTTGCGGAAAGCCTTAGAGTGGTAAATGAAACCATCG
CNE4  GAACAACAC.GAGAATATACTCGGTTGCGGAGAACCATAAAGTGGTAAATGAAACCATCG
CNE4  GAA.AAAAC.GAGAATATCCTCAGTTGCGGAAAGCCTTAGAGTGGAAAATGGAACCATCG
CNE4  GAAACGAGCCGAGAATATCCTCAGTTGTGGAAAGCCCTAGAGTGGTAAATGGGACCATCG
CNE4  GAATCGAGCCGAGAATGTCGTCAGTTGTGGAGAGCCTTAGAGTGGTAAATGGAACTATCG
CNE4  GAATCAAGCCGAGAAACTCCTCAGCTGTGGAAAGCCTTAGAGTGCTAAATGGAACCATCG
CNE4  GAATCGAGCCGAGA.TATCCTCAGTTGTGGAAAGCCTTAGAGTGGTAAATGGAACCATCG
CNE4  GAATCGAGCCGAGAATATCCTCATTTGCGGAAAGCCTTAGAGTGGTAAATGGAACCATCG

            120       130       140
CNE4  AAAAAGGAACCACCCGAGACCACTTCAA
CNE4  GAAAAGGAAGAACCCGAGACCACTTCAA
CNE4  GAAAAGGAAGAACCCGAGACCACTTCAA
CNE4  GAAACGGAAGAACCCGAGACCACTTCAA
CNE4  GAAAAGGAAGAACCCGAGACCACTTCAA
CNE4  GAAAAGGAAGAGCCCGCCACCACTTCAA
CNE4  GAAACGGAAGAACCCGAGACCACTT...
CNE4  GAAACGGAAGAACCAAGACCACTTCAA
CNE4  GAAATGGAAGAACCCGAGGCCACTTCAA
CNE4  GAAAAGGAAGAGCCCGACACCACTTCAA
CNE4  GAAACGGAAGAACTCGAGACCACTTCAA
CNE4  CGATTTT.....CCCCGGAACAATTCAT
CNE4  GAAAAGGAAGAACCCGAGACCACTTCAA
CNE4  GAAACGGAAGAACC.GAGACCACTTCAA
CNE4  GAAACGGAAGGACCCAATACCACTTCAA
CNE4  GAAACGGAAGAACCCAAGACCACTTCAA
CNE4  GAAACGGAAGAACTCGAGACCACTTCAA
CNE4  GAAATGGAAGAACCCGAGGCCACTTCAA
CNE4  GAAACGGAAGAACCCGAGACCACTTCAA
CNE4  GAAAAAGAAAAACCCGAGACCACTTCAA
```

Supplementary Fig. 5 Multiple Sequence alignment of CNE4. Nucleotides shaded red have ≥70% identity across the alignment. Image was produced with ESPript 3.0 (Robert, X. and Gouet,P. 2014).
